# Neuromodulation to prevent postoperative ileus: contemporary pathophysiology and emerging therapies

**DOI:** 10.1093/bjs/znag129

**Published:** 2026-11-10

**Authors:** Stephen J Chapman, Emily Pickworth, Emma Grant-Diaz, David G Jayne

**Affiliations:** Leeds Institute of Medical Research, University of Leeds, Leeds, UK; Leeds Institute of Medical Research, University of Leeds, Leeds, UK; Leeds Institute of Medical Research, University of Leeds, Leeds, UK; Leeds Institute of Medical Research, University of Leeds, Leeds, UK

## Postoperative ileus

Postoperative ileus is a debilitating complication occurring in 10–20% of all patients who undergo major abdominal surgery^1^. It is characterized by a transient impairment of intestinal function and presents clinically with vomiting, abdominal distension, and delayed defecatory function. The impact of ileus on recovery after surgery is profound. For patients, it increases the risk of serious complications, nutritional depletion, and prolonged hospital stay^2^. Ileus contributes significantly to compounded complications, leading to higher rates of end-organ dysfunction, reoperation, and hospital readmission^3^. For healthcare providers, ileus increases the economic burden of care and increases resource demand. Economic analyses report that it drives up total healthcare costs by over 66%, resulting from increased staff requirements as well as increased diagnostic, pharmacy, and hospital services^4,5^. As such, ileus prevention remains a major unmet challenge for the safe and effective delivery of surgical care across healthcare systems^6^.

Numerous clinical interventions to prevent ileus have been evaluated over the last 30 years but few have led to meaningful patient benefit. The most clinically effective have included the adoption of minimally invasive surgery, protocol-driven enhanced recovery, and multimodal opioid-sparing analgesia^1^. Other targeted interventions, such as sham enteral feeding (chewing gum) and prokinetic agents, have been evaluated extensively but few have shown sufficient promise for adoption^7,8^. Emerging strategies include neuromodulation, targeted anti-inflammatory agents, and non-pharmacological formulations (such as probiotics), but robust evidence to support their clinical effectiveness and adoption is still awaited. A major challenge in managing postoperative ileus is identifying interventions that are both biologically plausible as well as deliverable in clinical practice^9^. This requires a clear appreciation of the mechanisms that contribute to ileus, as well as the potential targets for intervention. This article outlines the current understanding of ileus, potential avenues for future patient benefit using neuromodulation, and the persisting uncertainties that may hold us back.

## Existing and contemporary understanding of postoperative ileus

Historically, postoperative ileus was considered to be an inevitable consequence of surgical stress, fluid imbalance, and opioid-induced side effects. It is well established that the physical trauma of incision and surgical manipulation triggers a transient inhibitory reflex that briefly disrupts normal motility^10^. Concurrently, rapid fluid administration induces intestinal oedema and impairs transit, while opioid-based analgesia further inhibits motility by activating enteric opioid receptors^11,12^. Although these factors undoubtedly contribute to postoperative ileus, they do not fully account for the prolonged period of gut dysfunction seen frequently after major abdominal surgery. Indeed, postoperative ileus persists despite the widespread adoption of Enhanced Recover After Surgery (ERAS) protocols, incorporating minimally invasive surgical techniques, judicious fluid management, and multimodal opioid-sparing analgesia^13^. We now recognize postoperative ileus as a complex, biphasic neuroimmune response, comprising a short-acting neurogenic phase triggered immediately upon skin incision, followed by a prolonged inflammatory phase in the subsequent days.

### Neurogenic phase of postoperative ileus

The immediate neurogenic phase of ileus is triggered by sympathetic reflexes in response to surgical trauma, such as skin incision and peritoneal traction. This mechanism is mediated via a spinal reflex arc. Afferent splanchnic nerves synapse in the spinal cord, activating an efferent sympathetic pathway which transiently inhibits intestinal motility^14^. At the cellular level, this is driven by noradrenaline binding to presynaptic α2-adrenergic receptors on myenteric neurons, suppressing excitatory neurotransmission by preventing the release of acetylcholine. Concurrently, non-adrenergic, non-cholinergic (NANC) neurons release nitric oxide and vasoactive intestinal peptide (VIP), which disrupt normal electrical signalling and prevent smooth muscle contraction. Crucially, this phase is self-limiting and offers only a narrow therapeutic window for intervention, as the inciting surgical stimuli cease after abdominal closure^15^.

### Later inflammatory mechanisms of postoperative ileus

The subsequent inflammatory phase commences 3–4 h after surgery, driven by a cascade of immune-mediated events in which the activation of intestinal macrophages plays a key role^15^. These macrophages are stimulated by damage-associated molecular patterns (DAMPs) released during manual intestinal handling, alongside pathogen-associated molecular patterns (PAMPs) such as lipopolysaccharide (LPS). The presence of PAMPs is facilitated by activated mast cells, which increase mucosal permeability and permit the translocation of luminal bacteria and their products into the systemic circulation^16^. Upon binding DAMPs and PAMPs, intestinal macrophages produce pro-inflammatory cytokines (such as tumour necrosis factor α (TNFα), interleukin (IL) 1β, and IL6) and chemokines (such as monocyte chemoattractant protein-1 (MCP-1)). These upregulate endothelial adhesion molecules, notably intercellular adhesion molecule-1 (ICAM-1), which promote the recruitment and extravasation of leucocytes into the intestinal muscularis. Here, infiltrating leucocytes trigger inducible nitric oxide synthase (iNOS) and cyclooxygenase-2 (COX-2), producing nitric oxide and prostaglandins which profoundly impair local smooth muscle contractility^14^. Ultimately, generalized ileus develops throughout the gut as proinflammatory mediators and their metabolites activate inhibitory neural pathways, disrupting neuromuscular function in distant, unhandled segments of the gut.

## The cholinergic anti-inflammatory pathway as a target to prevent postoperative ileus

The cholinergic anti-inflammatory pathway was first described in 2000 as an endogenous anti-inflammatory system capable of preventing sepsis^17^. In preclinical models, stimulating the vagus nerve prevented endotoxin-induced septic shock by inhibiting the expression of proinflammatory cytokines from splenic macrophages. This was mediated by nicotinic receptors in the spleen in response to acetylcholine released from vagal efferent nerve endings. The concept was later extended to the gut, where it was established that intestinal handling and subsequent inflammation triggered a vago-vagal anti-inflammatory reflex. It was shown that vagal afferent fibres activate vagal centres in the brainstem (such as the nucleus of the solitary tract and the dorsal motor nucleus of the vagus) in response to inflammation, which in turn stimulate efferent vagal fibres targeting the inflamed tissue^18^ (*Fig. 1*). Binding of acetylcholine to the α7 subunit of nicotinic receptors (α7nAChR) on intestinal macrophages was shown to trigger an intracellular cascade involving the JAK2–STAT3 signalling pathway. This inhibited macrophage activation, suppressed the release of inflammatory cytokines, reduced the recruitment of leucocytes to the muscularis, and prevented ileus^19^.

**Figure 1:**
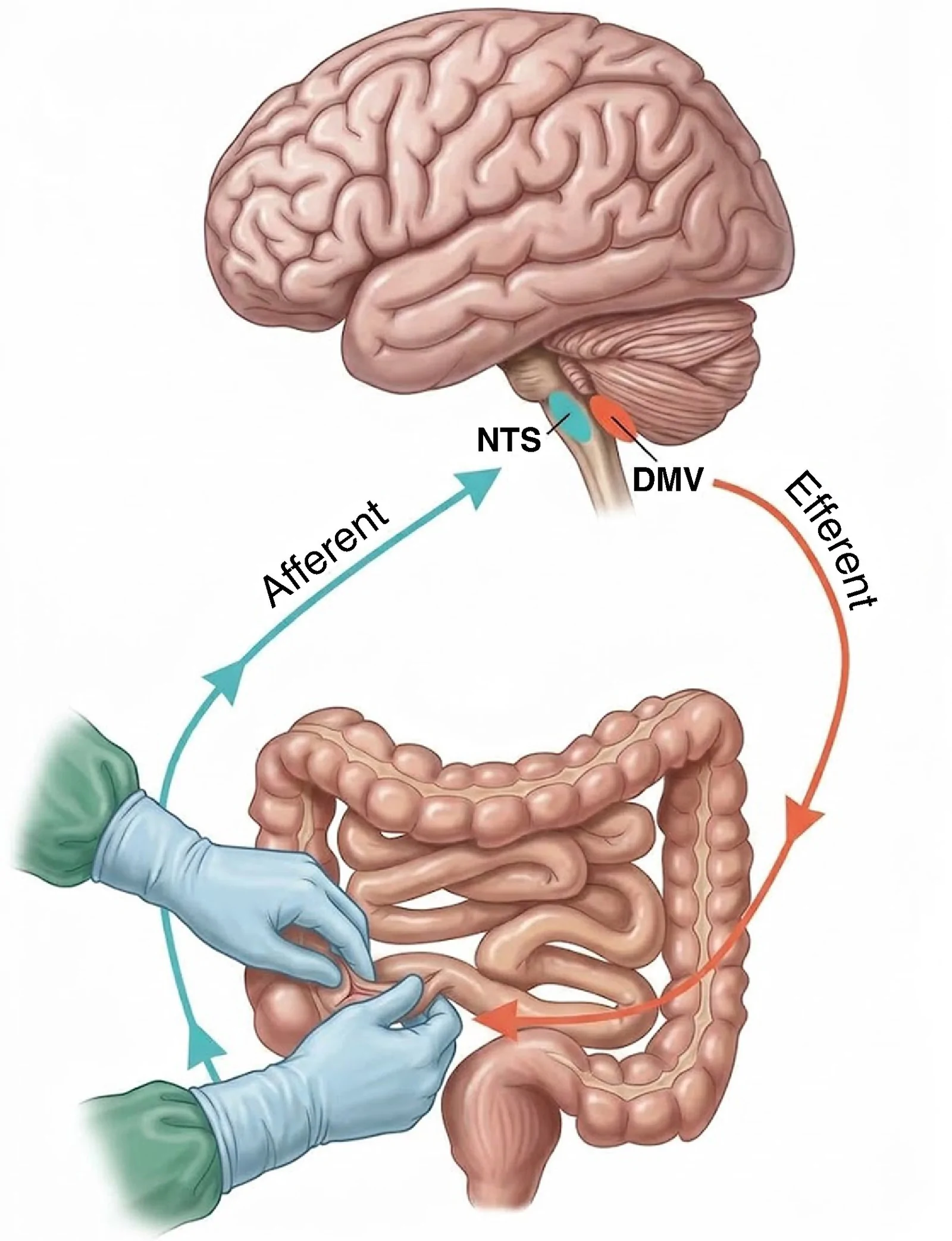
**Afferent and efferent components of the cholinergic anti-inflammatory reflex**DMV: Dorsal motor nucleus of the vagus nerve; NTS: nucleus of the solitary tract.

Preclinical studies using a standardized model of ileus provide consistent evidence in support of a cholinergic anti-inflammatory pathway in the gut and its role after surgery. In a study of the experimental nAChR agonist AR-R17779, it was shown that delayed gastric emptying and inflammatory cell infiltration were significantly reduced in mice treated with the active agent compared to a saline control^20^. In another study of electrical vagus nerve stimulation (VNS), it was shown that active VNS significantly reduced intestinal inflammation and improved transit. Unlike previous observations in sepsis, the effects of VNS were entirely independent of the spleen, instead requiring α7nAChR receptors to be present on intestinal macrophages^18^. Notably, other studies have shown that division of the vagus nerve (vagotomy) increases intestinal inflammation by eliminating vagal signalling. In preclinical models, eliminating this neuro-immune reflex exacerbates the impairment of intestinal smooth muscle after surgery, leading to a more prolonged delay in transit^21^.

## Neuromodulation therapies for postoperative ileus

Neuromodulation for postoperative ileus has emerged as an alternative to conventional pharmacotherapy. It leverages endogenous neuroimmune reflexes, such as the vagal cholinergic anti-inflammatory pathway, by stimulating specific peripheral nerves. The mechanism of electrical neuromodulation is based on the principles of actional potential, a rapid sequence of changes in voltage across a cell membrane leading to propagating cycles of depolarization and repolarization. The extrinsic activation of nerve cells by an external pulse depends on several properties, including the electrode-to-nerve distance, the degree of myelination, and the diameter of nerve cells. Shorter distances from the external pulse, greater myelination, and larger target nerve cells all increase the likelihood of depolarization^22^. Applying these principles, a number of invasive and non-invasive approaches to reduce postoperative ileus using neuromodulation strategies have been explored previously.

### Invasive vagus nerve stimulation

Early studies explored the efficacy of invasive vagus nerve stimulation (iVNS) in mouse and rat models of ileus. In mice undergoing laparotomy, stimulation of the left cervical vagus nerve prevented intestinal manipulation-induced gastroparesis (gastric retention: 43.0 ± 6.7% *versus* 25.2 ± 3.2%; *P* < 0.050) 24 h after surgery^19^. Similarly, in a rat model of sleeve gastrectomy, subdiaphragmatic iVNS accelerated the time to first drinking (41.2 ± 8.1 *versus* 89.1 ± 15.1 h; *P* = 0.010) and first defaecation (38.8 ± 7.1 *versus* 124.4 ± 32.1 h; *P* = 0.020), while also improving gastric emptying at 72 h (emptying: 79.4 ± 4.9% *versus* 60.8 ± 5.7%; *P* = 0.010)^23^. These benefits were further corroborated in mice, where abdominal iVNS improved intestinal transit (geometric centre: 7.8 ± 0.6 *versus* 5.1 ± 0.2; *P* < 0.010) and reduced intestinal inflammation (35 ± 7 *versus* 80 ± 8 myeloperoxidase (MPO)-positive cells/field; *P* < 0.050)^24^. Notably, the same study demonstrated that abdominal iVNS in humans significantly decreased whole-blood IL-6 and IL-8 levels (*P* < 0.050). However, given the clinical challenges of performing invasive stimulation in humans, the majority of later research pivoted towards non-invasive techniques.

### Transcutaneous vagus nerve stimulation

Transcutaneous vagus nerve stimulation (tVNS) can be performed using cervical and auricular approaches. Cervical tVNS typically involves a self-administered device applied to the neck over the carotid surface landmark. An unpowered study of 40 patients revealed that twice-daily stimulation using cervical tVNS after colorectal surgery shortened the time to first flatus (2.35 ± 1.32 *versus* 1.65 ± 0.880) and solid diet (2.18 ± 2.21 *versus* 1.75 ± 0.910) compared to sham control^25^. A subsequent feasibility study confirmed the safety of cervical tVNS in 100 patients, as well as demonstrating high patient acceptability driven prominently by its self-administered form factor^26^. Other studies have explored auricular tVNS, targeting the auricular branch of the vagus nerve using electrodes attached to the tragus or cymba concha of the ear. In a study of 14 patients undergoing laparotomy, auricular tVNS after surgery increased pyloric action potential amplitudes compared to control (0.25 ± 0.19 *versus* 0.19 ± 0.14 mV; *P* < 0.005)^27^. In an RCT of 134 patients undergoing minimally invasive colorectal surgery, auricular tVNS for 20 min before anaesthesia reduced the incidence of ileus compared to control (6.3 *versus* 20.0%; *P* = 0.022)^28^. A further RCT of 22 patients showed that daily auricular tVNS reduced the time to flatus (62.0 ± 21.1 h *versus* 90.2 ± 27.1)^29^. In all studies, the safety of tVNS was favourable, with no reports of serious adverse events and other reports limited to mild discomfort at the stimulation site.

### Transcutaneous posterior tibial nerve stimulation

Posterior tibial nerve stimulation (PTNS) is well established for the treatment of overactive bladder and faecal incontinence. As a preventative therapy for ileus, it is hypothesized that peripheral stimulation at the ankle indirectly modulates the pelvic plexus, increasing parasympathetic outflow whilst dampening sympathetic tone. In a single-centre RCT of 40 patients undergoing colectomy, PTNS administered three times daily neither shortened the time to first bowel function (3.6 *versus* 3.1 days; *P* = 0.600) or reduced the incidence of ileus (35% *versus* 18%; *P* = 0.420)^30^. Similarly, a larger RCT of 340 patients undergoing colectomy demonstrated that PTNS did not shorten the time to flatus or stool compared to sham stimulation. Nevertheless, in exploratory analyses, patients receiving PTNS following right-side colectomy had a significantly shorter time to first flatus (33.0 ± 20.5 *versus* 46.0 ± 27.8 h; *P* = 0.040)^31^.

### Electroacupuncture and transcutaneous electrical acupoint stimulation

Electroacupuncture (EA) and transcutaneous electrical acupoint stimulation (TEAS) have roots in traditional Chinese medicine. Both target specific somatic acupoints (most commonly Zusanli ST36 and Neiguan PC6), but EA requires the insertion of fine acupuncture needles whereas TEAS uses non-invasive adhesive electrodes. The proposed mechanism involves the cholinergic anti-inflammatory pathway and is supported by robust evidence from multiple RCTs. In a meta-analysis of 16 studies (1562 patients), EA reduced the time to first flatus (mean difference −10.1 h, 95% c.i.: −12.27 to −7.94, *P* < 0.001) and reduced the time taken to tolerate solid food (−17.21 h, 95% c.i.: −29.29 to −5.13, *P* = 0.005)^32^. The need for specialist practitioners, however, limits its clinical adoption in Western healthcare systems. TEAS offers a non-invasive alternative, although current evidence to support its clinical efficacy is conflicted. In an RCT of 610 patients undergoing colorectal surgery, lower limb TEAS (ST36, ST37, and SP6) significantly reduced ileus (32.3% *versus* 41.0%; *P* = 0.0260)^33^. Conversely, an RCT of 441 patients undergoing mixed abdominal surgery found that combined upper- and lower-limb TEAS (ST36, SP6, PC6, LI4) did not accelerate the recovery of bowel function^34^. It is plausible that the efficacy of TEAS depends on the specific acupoints targeted.

## Current gaps and future directions

The evidence supporting neuromodulation therapies for the prevention of postoperative ileus varies substantially by modality. Although data for PTNS and TEAS remain inconclusive, VNS shows promise for accelerating the return of bowel function^25,27–30^. Furthermore, the clinical feasibility of VNS is well-established, alongside a clear understanding of the barriers and facilitators to its implementation in practice^26^. In contrast, EA is supported by robust RCTs demonstrating improved bowel recovery and reduced length of stay^32^. However, because these trials were conducted exclusively in China, where traditional therapies are integrated into routine care, the generalizability and deployability of these findings to international healthcare settings is uncertain. In the most part, the evidence for neuromodulation therapies to prevent or reduce ileus remains at an early but promising stage.

Although the current evidence for neuromodulation establishes a foundation for later-phase evaluations, critical methodological limitations persist for research on postoperative ileus. Outcome assessment remains highly heterogenous, with over 73 distinct endpoints reported in existing literature^35^. Recent efforts to address this include the development of an agreed core outcome set and a disease-specific patient reported outcome measure^36,37^, both seeking to improve patient-centricity in future trials. Another limitation is the lack of adequately reported diversity data in previous studies. This precludes an evaluation of treatment effects across specific groups and communities, underscoring the need for an agreed core descriptor set^38^. Finally, the evaluation of neuromodulation therapies in any setting is challenging due to well-established placebo effects associated with electrical stimulation interventions. Sham therapies produce clinically and statistically significant improvements in outcomes, meaning that robust sham-controlled designs are essential^39^. In the case of VNS, this may comprise an anatomical sham (active stimulation at a sham site), a parameter sham (subtherapeutic stimulation at the correct site), or a device sham (inactive device at the correct site).

Future research must identify and evaluate interventions for postoperative ileus more efficiently. At present, many late-phase trials yield disappointing results due to a lack of early-phase evaluation to support a definitive assessment. This increases the risk of selecting non-efficacious interventions, missed opportunities to identify patient groups with the highest chance of benefit, and uncertain interactions between clinical co-interventions. The latter additionally leads to missed opportunities to explore cumulative benefits of concurrent treatments. Establishing an early-phase platform or adaptive trial design could bridge this gap. Platform and adaptive designs are disease-focused clinical studies evaluating multiple treatments simultaneously using a master protocol and shared infrastructure. In the context of ileus, this could enable investigators to graduate efficacious interventions to targeted late-phase clinical trials while rapidly discarding those which lack efficacy. Furthermore, establishing a master protocol could improve the uptake of ileus-specific evidence-based method frameworks, such as an agreed core outcome set and patient-reported outcome measures (PROMs). On the other hand, platform and adaptive trial designs are methodologically and logistically complex. A community-wide collaboration is required to deliver this ambition, involving clinical, academic, regulatory, industry, policy, and public partners. The reward for patients, healthcare professionals, health systems, and economies could be an end to ileus and its sequelae within the next 10 years.

## Data Availability

No primary data are available.

## Data sharing

All data are reported in the manuscript.
